# Radiographic Critical Shoulder Angle Combined With Greater Tuberosity Irregularities and Patient Age Accurately Predicts Rotator Cuff Pathology

**DOI:** 10.7759/cureus.96569

**Published:** 2025-11-11

**Authors:** Sharo Naqar, Islam Hamarsheh, Jae Rhee, Anestis Iossifidis

**Affiliations:** 1 Trauma and Orthopaedics, Croydon University Hospital, London, GBR

**Keywords:** critical shoulder angle, rotator cuff pathology, rotator cuff tears, shoulder girdle, subacromial impingement

## Abstract

Introduction

The combination of a high critical shoulder angle (CSA), the presence of radiographic greater tuberosity (GT) irregularities, and the patient’s age can aid surgeons in diagnosing rotator cuff tears (RCTs). This study aims to assess the usefulness of these simple radiographic measures in a clinical outpatient setting. We hypothesise that a high CSA, together with the presence of GT irregularities and the patient's age, is associated with a high probability of RCT.

Methods

Radiographs of 150 patients were examined retrospectively. The final cohort consisted of 150 patients: 50 with normal shoulders (group A), diagnosed clinically and radiologically using MRI scan, 50 with subacromial impingement (SAI) (group B), and 50 with RCTs (group C). The CSA was measured electronically on true anteroposterior (AP) shoulder radiographs using a picture archiving and communication system (PACS). The presence of radiographic GT irregularities was also recorded according to the following classification: 0 = normal, 1 = sclerosis, and 2 = irregularity or a bony chip. A senior and one training surgeon took measurements. Mean CSAs were used for analysis, as confirmed by a high interobserver agreement, as determined by an interclass correlation coefficient (ICC) calculation. The p-value was set at 0.05.

Results

The mean CSA for groups A, B, and C was 32.4 (standard deviation (SD) ± 1.5), 34.8 (SD ± 3.2), and 39.3 (SD ± 3.4), respectively. Statistical analysis showed excellent discrimination between normal and cuff pathology, with an area under the curve (AUC) of 0.881 and an optimal CSA cutoff of 34.6° (sensitivity, 74%; specificity, 92%; accuracy, 80%). We then sought a cutoff between SAI and cuff tears and found 36.9 to be significant (AUC 0.848). CSA of ≥36.9° can differentiate between SAI and cuff tears (sensitivity, 78%; specificity, 76%; accuracy, 77%). Combining the CSA of ≥34.8° with GT of 2 can outstandingly discriminate a cuff tear (AUC 0.920, up from 0.848 with CSA alone). The addition of age can generate further improvement, with an AUC of 0.953 (sensitivity, 80%; specificity, 96%; accuracy, 88%).

Conclusion

This study shows that at age 40 years, RCT was predicted if CSA ≥ 44.9° (GT = 1) or ≥41.0° (GT = 2); at age 50 years, thresholds were CSA ≥ 42.0° (GT = 1) or ≥38.0° (GT = 2); and at age 60 years, CSA ≥ 39.1° (GT = 1) or ≥35.1° (GT = 2). Applying these age-banded thresholds yielded a sensitivity of 80%, a specificity of 96%, and an overall accuracy of 88%. Therefore, a simple radiograph-based model combining CSA, GT morphology, and age may allow clinicians to stratify patients at the first point of contact, streamlining diagnostic pathways, reducing unnecessary advanced imaging, and guiding early intervention strategies.

## Introduction

Rotator cuff tears (RCTs) and osteoarthritis (OA) of the glenohumeral joint are common yet almost mutually exclusive pathologies, and the reasons for this remain poorly understood [1]. Earlier research focused on various measurements, including the Bigliani classification (types I-III), Aoki’s acromial slope, Nyffeler’s acromial index, and Banas’ lateral acromial angle, to aid in diagnosing shoulder pathologies [2]. These measures often varied with radiographic quality or degenerative changes, limiting their reliability. To overcome these limitations, the critical shoulder angle (CSA) was introduced by Moor et al. and is thought to influence the risk of degenerative RCTs, with a range of 30° to 35° generally considered to be a ‘favourable range’ [1].

Studies have found that individuals with degenerative RCTs have significantly larger CSAs (≥35°) compared to those with asymptomatic shoulders [1,2]. Measurement of accurate CSA is highly dependent on proper anteroposterior (AP) radiographs, and deviations as little as 5° anteversion alter results [3]. Gerber et al. confirmed that a large CSA alters glenohumeral biomechanics, such that it could induce supraspinatus overload, and that a low CSA increases the load of the humeral head on the glenoid [4,5].

However, a meta-analysis showed that while the CSA can be reliably measured, the difference in the CSA between cases and controls varied from very large to almost no difference, and it is difficult to understand the strength and association between the CSA and RCT with the current evidence [6]. A meta-regression analysis revealed that the sensitivity of CSA could be higher for differentiating full-thickness RCTs from normal patients [7].

Despite robust evidence backing CSA as a strong parameter, relying on a single parameter can pose limitations, including radiograph quality, borderline CSA values, and heterogeneity. Other markers, such as greater tuberosity (GT) morphology, have not been systematically integrated with CSA.

This study aimed to evaluate whether combining CSA with GT morphology and age improves the prediction of RCTs beyond CSA alone. We hypothesised that this combined model would enhance diagnostic accuracy and provide a more robust, radiograph-based risk stratification tool.

## Materials and methods

This retrospective, single-centre study was conducted at our South-West London university hospital and included patients treated between January 2014 and March 2025. Eligible patients were those diagnosed with subacromial impingement (SAI), confirmed by arthroscopy, and those who underwent an arthroscopic rotator cuff repair confirmed to have an RCT, clinically, radiologically, and by arthroscopy, during the study period. Patients were selected primarily based on the presence of suitable shoulder radiographs, and those without suitable AP radiographs were excluded. A suitable AP radiograph was a true, standard AP view, often referred to as the Grashey view, which minimised scapular positioning errors. The final cohort consisted of 150 patients: 50 with normal shoulders (group A), diagnosed clinically and radiologically using MRI, 50 with SAI (group B), and 50 with RCTs (group C).

**Figure 1:**
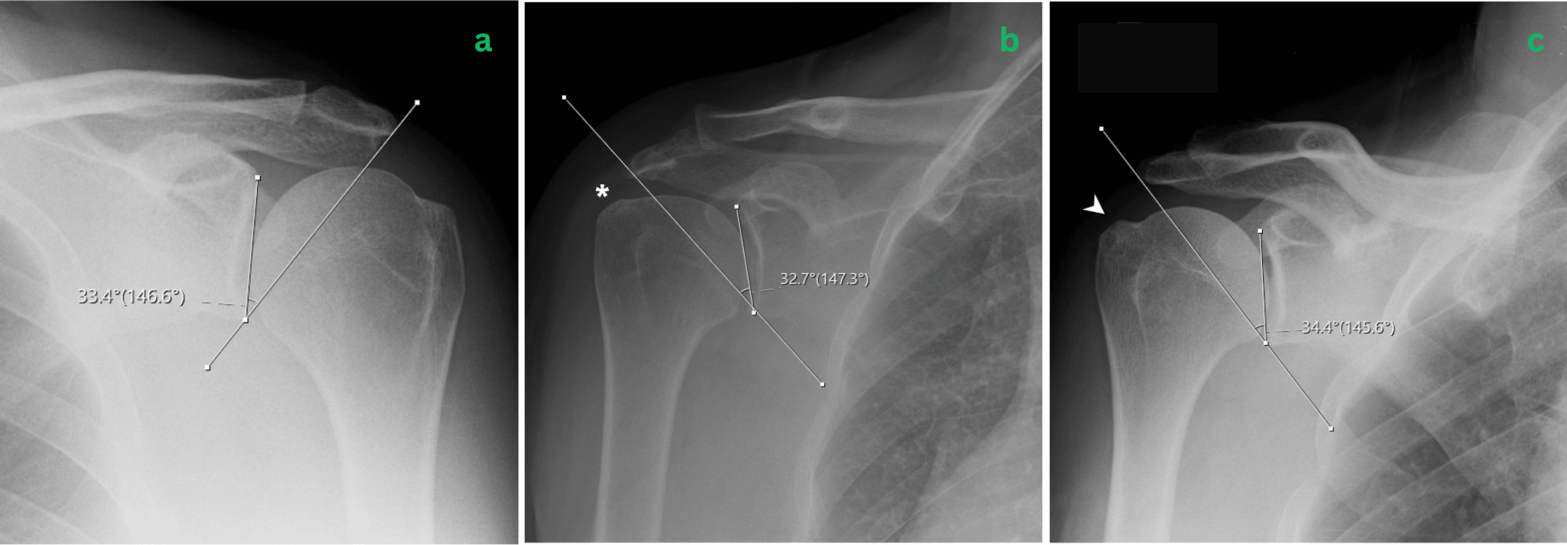
Representative shoulder radiographs from each study cohort. (a) Normal radiograph of a patient from group A. (b) Radiograph of a patient from group B (subacromial impingement) demonstrating greater tuberosity (GT) sclerosis (*). (c) Radiograph of a patient from group C (rotator cuff tear) showing GT irregularity (white arrowhead).

Ethical approval was not required as all data were anonymised before analysis. Patient demographics (age, gender, and date of surgery) were collected from hospital medical records for each patient. Radiographic assessment focused on CSA and GT morphology, measured electronically on true AP shoulder radiographs using a picture archiving and communication system (PACS). GT morphology was categorised into three categories: 0 = normal, 1 = sclerosis, and 2 = irregularity or bone chip (Figure 1). All radiographs were independently reviewed by two blinded observers, a consultant shoulder surgeon and a specialty registrar.

Interobserver reliability for CSA was assessed using the intraclass correlation coefficient, and Bland-Altman analysis was performed to evaluate both systematic bias (mean difference) and the limits of agreement between observers, providing a robust measure of reliability in continuous radiographic measurements. Given the excellent reliability, the mean CSA value was used for subsequent analysis.

Statistical analysis was performed using IBM SPSS Statistics (version 26, IBM Corp., Armonk, NY, USA). Descriptive statistics were used to summarise demographic variables. Comparisons of CSA across diagnostic categories (normal, SAI, and cuff tear) were performed using the Kruskal-Wallis test, as the data did not follow a normal distribution completely. Where overall differences were significant, pairwise group comparisons were conducted using the Mann-Whitney U test as a post hoc procedure, providing a robust method for assessing differences between independent groups when the assumption of normality is violated. GT distributions across groups were compared using the chi-squared test of independence.

Binary logistic regression modelling was then undertaken in two stages. First, pathology (0 = normal, 1 = SAI/cuff tear) was regressed on CSA as the sole predictor. Model performance was assessed by the area under the curve (AUC), McFadden’s pseudo-R², and operating characteristics at classification thresholds of 0.5 and 0.7. Optimal CSA cutoff values were derived using receiver operating characteristic (ROC) analysis and the Youden index, with thresholds translated into clinically interpretable angular values.

Within the pathology cohort (SAI and cuff tear), binary logistic regression was used to identify predictors of cuff tear. CSA was first included alone, followed by GT grade as a categorical covariate and finally age as a continuous covariate. Model performance was again evaluated using AUC, McFadden’s pseudo-R², and the Youden index to derive optimal thresholds. In addition, stratified CSA thresholds by GT and age were explored, and simplified decision rules (e.g., CSA-only threshold, CSA + GT ‘OR’ rule) were tested for clinical applicability. To assess model reliability, calibration was evaluated using calibration plots. A two-sided p-value < 0.05 was considered statistically significant. Test statistics (H values for the Kruskal-Wallis test and U values for the Mann-Whitney test) are reported alongside p-values to ensure transparency and reproducibility.

## Results

A total of 150 patients were included in the analysis, divided evenly across the three groups: normal (n = 50, 33.3%), SAI (n = 50, 33.3%), and RCT (n = 50, 33.3%). The overall cohort comprised 80 men (53.3%) and 70 women (46.7%). The characteristics of the participant are shown in Table 1.

**Table 1:** Demographic characteristics of patients across the three diagnostic groups (normal, SAI, and RCT). Values are presented as mean (m) ± standard deviation (SD) or n (%). SAI: subacromial impingement; RCT: rotator cuff tear.

Variable	Normal (n = 50)	SAI (n = 50)	RCT (n = 50)	Total (n = 150)
Age, m ± SD	46.9 ± 13.7	51.7 ± 9.0	62.2 ± 9.2	53.6 ± 12.5
Gender, n (%)				
Male	33 (66.0)	22 (44.0)	25 (50.0)	80 (53.3)
Female	17 (34.0)	28 (56.0)	25 (50.0)	70 (46.7)

**Figure 2:**
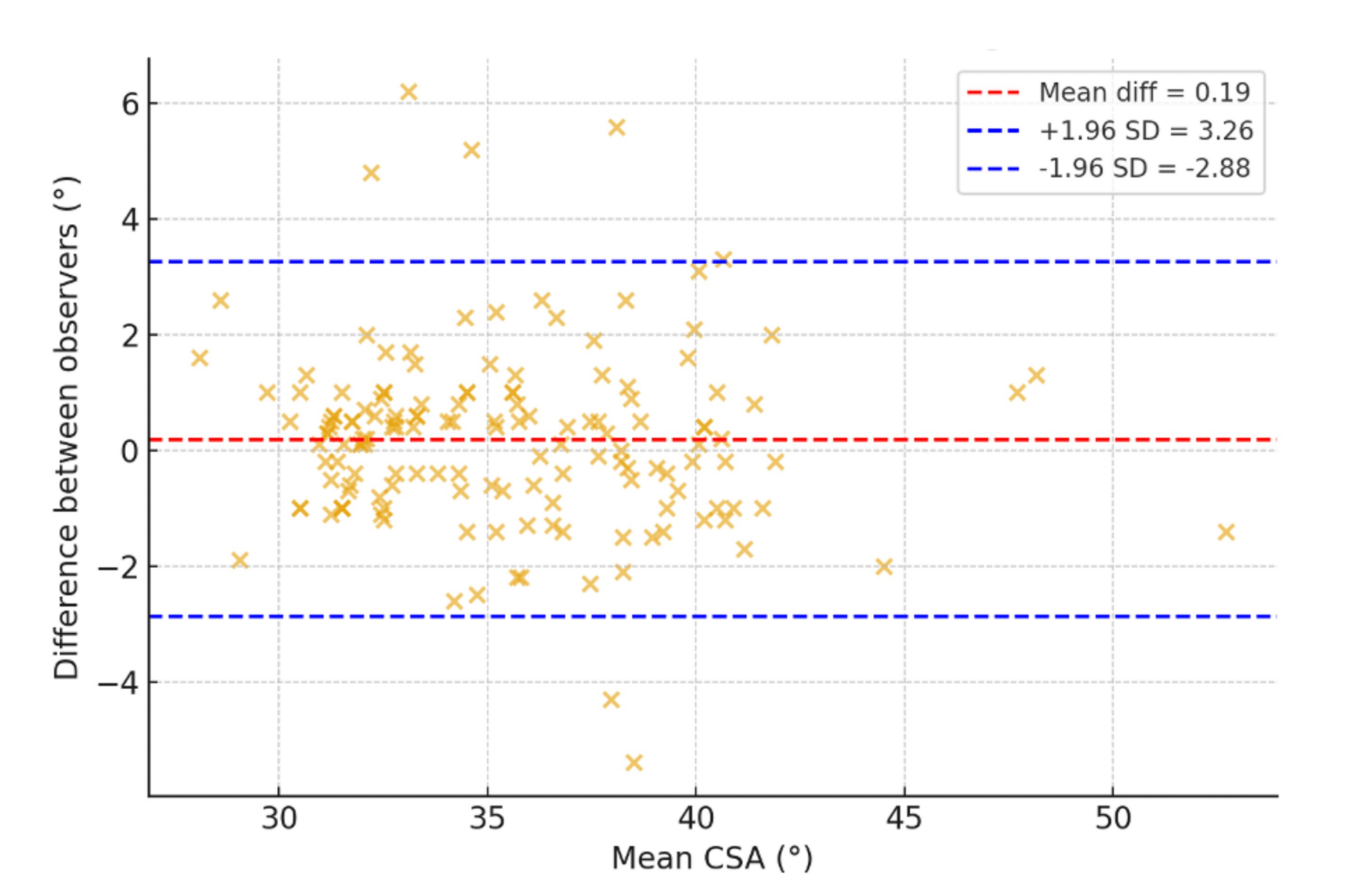
Bland-Altman plot for CSA measurements. SD: standard deviation; CSA: critical shoulder angle.

CSA was measured independently by two blinded observers on AP shoulder radiographs. Interobserver reliability was excellent, with an interclass correlation coefficient (ICC) of 0.93. Bland-Altman analysis revealed a slight bias of M = 0.19° (95% CI, −0.06° to 0.45°), with 95% limits of agreement ranging from −2.88° to 3.26° (standard deviation (SD) = 1.57°), indicating excellent agreement and confirming high reproducibility (Figure 2). In view of the superb agreement, the average CSA from both observers was used for subsequent analysis.

**Figure 3:**
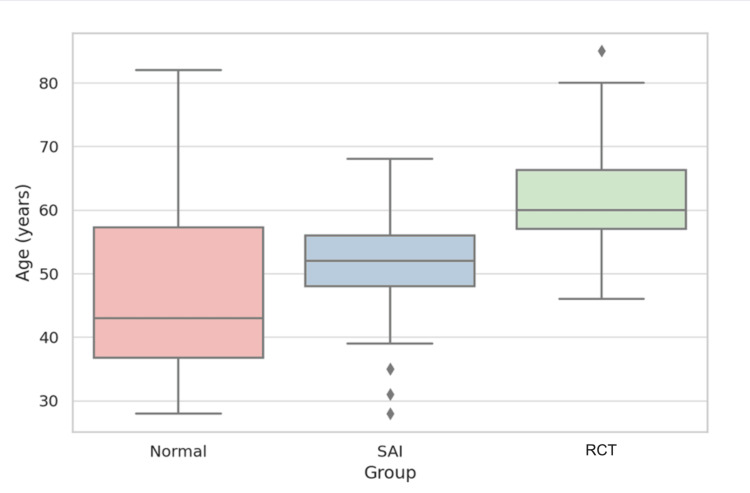
Boxplot of age distribution across diagnostic groups. Age increased progressively from the normal to the RCT group, with minimal overlap between distributions, as shown by the spread of medians and interquartile ranges. Kruskal-Wallis test: H(2) = 39.07, p < 0.001. SAI: subacromial impingement; RCT: rotator cuff tear.

The overall mean age of the study sample was 53.6 ± 12.5 years (median 55.0; range 28-85). Patients in the normal group had a mean age of 46.9 ± 13.7 years (median, 43.5 years); those with SAI had a mean age of 51.7 ± 9.0 years (median, 52.5 years); and those with RCT had a mean age of 62.2 ± 9.2 years (median, 60.5 years). Figure 3 illustrates the distribution of age across diagnostic groups. The Kruskal-Wallis test confirmed a statistically significant difference in age distribution among the three groups, H(2) = 39.07, p < 0.001.

**Figure 4:**
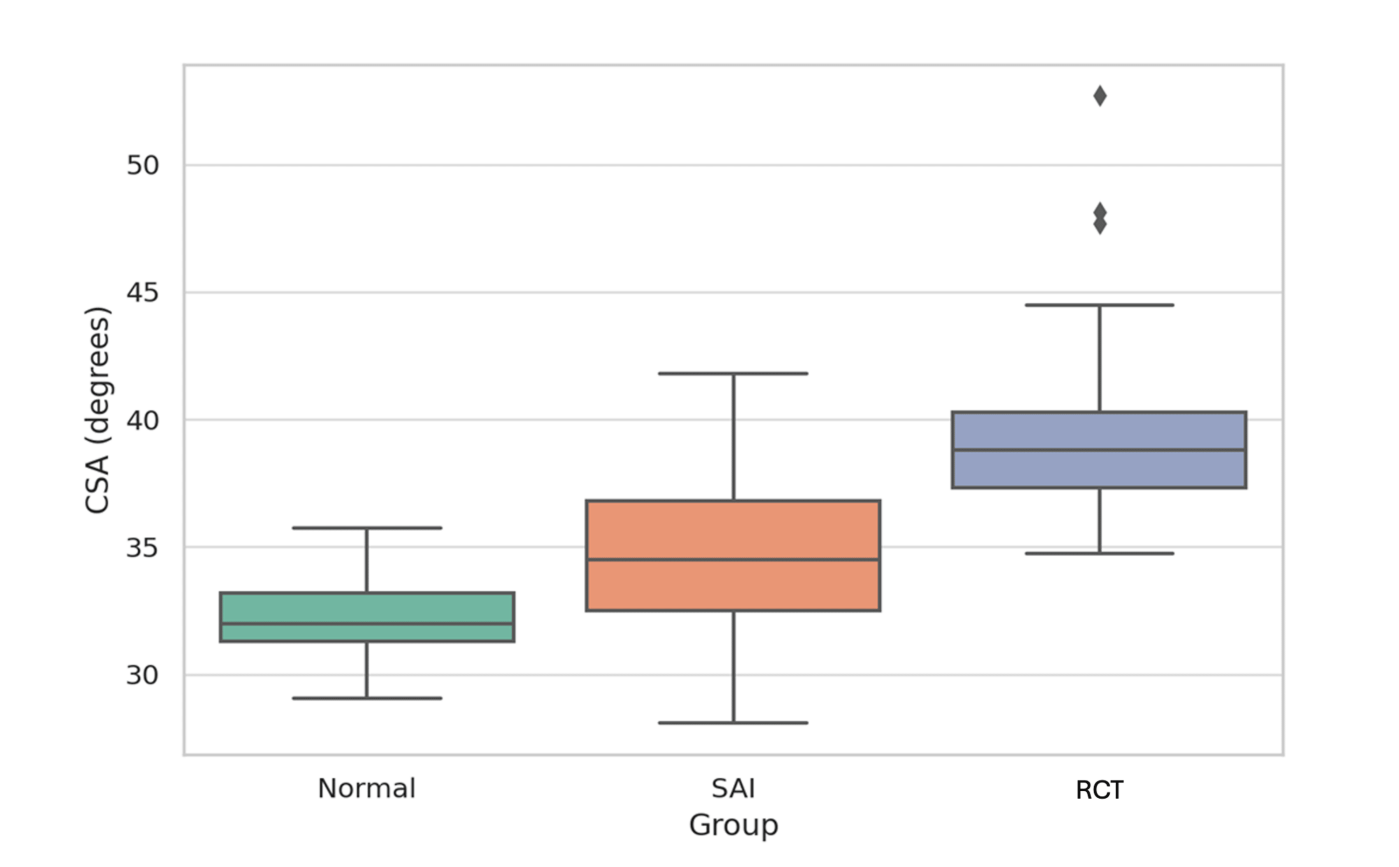
Boxplot of critical shoulder angle (CSA) across diagnostic groups. CSA increased progressively from the normal to the RCT group, with minimal overlap between distributions, as shown by the spread of medians and interquartile ranges. Kruskal-Wallis test: H(2) = 85.85, p < 0.001. Mann-Whitney U post hoc comparisons: normal vs. SAI (U = 580.5, p < 0.001); normal vs. RCT (U = 15.0, p < 0.001); SAI vs. RCT (U = 379.5, p < 0.001). RCT: rotator cuff tear; SAI: subacromial impingement.

The overall mean CSA for the entire cohort was 35.5° ± 4.0. Across diagnostic categories, mean CSA increased progressively from the normal (32.3° ± 1.5) to the SAI (34.8° ± 3.2) and the RCT (39.3° ± 3.4) groups. Table 2 summarises these data, and Figure 4 illustrates the distribution of CSA values, showing a clear rightward shift in median and range with minimal overlap between groups. A Kruskal-Wallis test confirmed a significant difference in CSA among diagnostic groups (H(2) = 85.85, p < 0.001), and Mann-Whitney U post hoc tests demonstrated significant pairwise differences: normal vs. SAI (U = 580.5, p < 0.001), normal vs. RCT (U = 15.0, p < 0.001), and SAI vs. RCT (U = 379.5, p < 0.001).

**Table 2:** Critical shoulder angle (CSA, °) across groups. Kruskal-Wallis test: H(2) = 85.85, p < 0.001; post hoc Mann-Whitney U values for pairwise comparisons are reported in the text. SAI: subacromial impingement; RCT: rotator cuff tear; SD: standard deviation.

Group	Mean ± SD	Median	Min	Max	n
Normal	32.31 ± 1.46	31.98	29.05	35.75	50
SAI	34.82 ± 3.15	34.48	28.10	41.80	50
RCT	39.29 ± 3.35	38.80	34.75	52.70	50

GT morphology differed significantly across diagnostic groups. All patients in the normal group had GT grade 0. In the SAI group, most patients demonstrated a GT grade of 1, with a smaller proportion showing a grade of 2. In contrast, within the RCT group, 32% exhibited sclerosis (grade 1), and the majority (68%) demonstrated irregularity or a bone chip (grade 2), as shown in Table 3. The chi-squared test confirmed a strong association between GT morphology grade and diagnostic group (χ² = 213.05, p < 0.0001).

**Table 3:** Greater tuberosity (GT) irregularities across groups. Chi-squared test: χ² = 213.05, p < 0.0001. SAI: subacromial impingement; RCT: rotator cuff tear.

GT grade	Normal (n = 50)	SAI (n = 50)	RCT (n = 50)	Total (n = 150)
0 (normal)	50 (100%)	0 (0.0%)	0 (0.0%)	50
1 (sclerosis)	0 (0.0%)	42 (84.0%)	16 (32.0%)	58
2 (irregularity/bone chip)	0 (0.0%)	8 (16.0%)	34 (68.0%)	42
Total	50	50	50	150

Regression analysis and predictive performance

**Figure 5:**
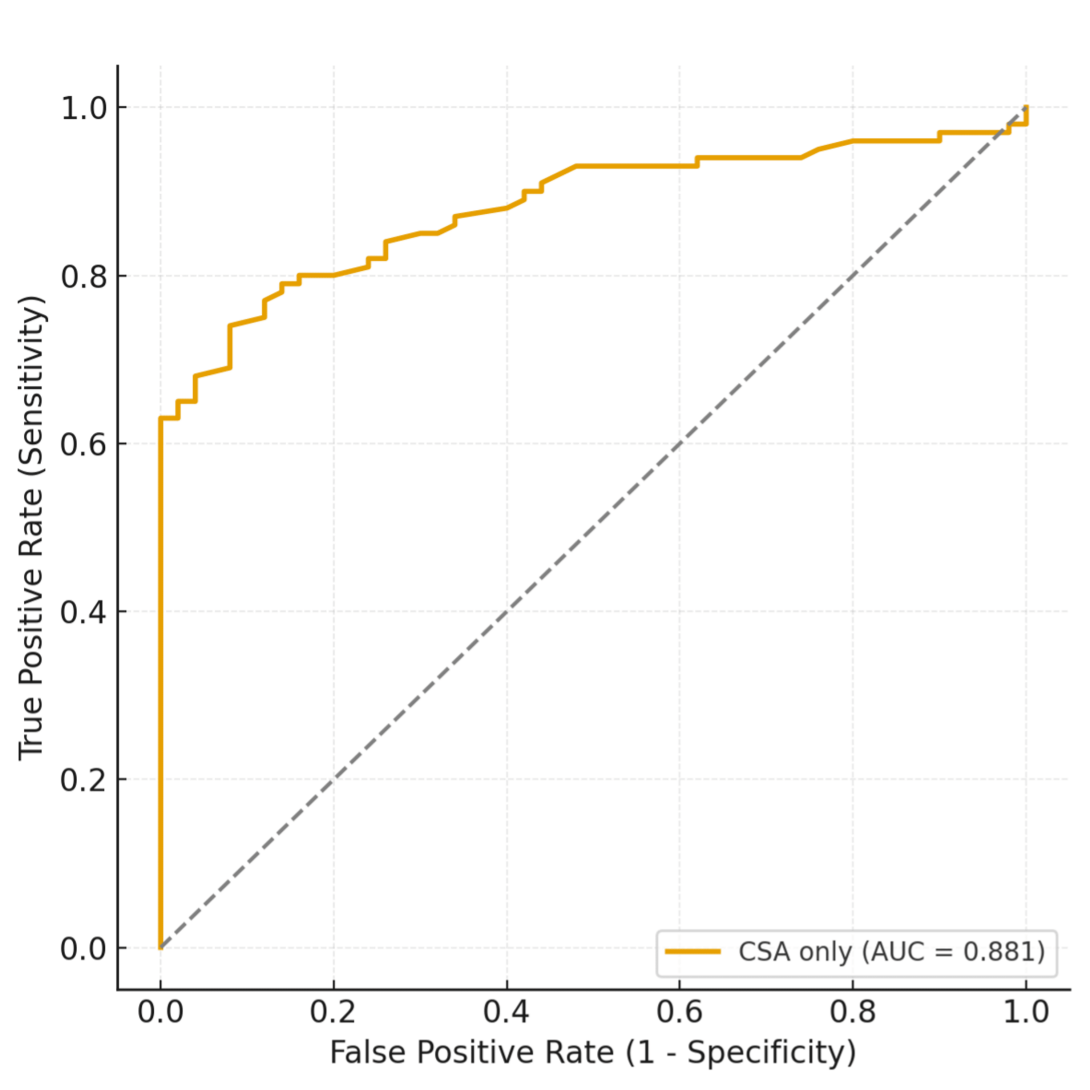
Receiver operating characteristic (ROC) curve for CSA in differentiating normal from pathological shoulders. The model demonstrated good discrimination with an AUC of 0.88. The optimal cutoff for CSA (34.6°) corresponded to a sensitivity of 74% and a specificity of 92%. CSA: critical shoulder angle; AUC: area under the curve.

In univariate logistic regression, CSA was a highly significant predictor of pathology. For each additional degree of CSA increase, the odds of having pathology increased by 83% (OR 1.83, 95% CI 1.49-2.25, p < 0.001). The model demonstrated good discrimination with an AUC of 0.88 and McFadden’s pseudo-R^2^ of 0.36, reflecting the strong explanatory power of a single-predictor clinical model (Figure 5).

At a classification threshold of 0.50, the model achieved an overall accuracy of 80.7%, a sensitivity of 84%, and a specificity of 74%. At a stricter threshold (0.70), accuracy was 80.0%, with higher specificity (88%) but lower sensitivity (76%).

The optimal cutoff for CSA to differentiate normal from pathological shoulders was identified using ROC analysis. The Youden index (sensitivity + specificity - 1) was applied to determine the threshold that maximised combined sensitivity and specificity. The corresponding predicted probability was then translated back into CSA degrees using the logistic regression equation, yielding a clinically interpretable cutoff. Table 4 summarises the diagnostic performance of CSA at different angular thresholds.

**Table 4:** CSA cutoffs and diagnostic performance - normal vs. pathology. CSA: critical shoulder angle.

CSA cutoff (°)	Sensitivity	Specificity	Accuracy
32.0	0.93	0.50	0.79
34.0	0.80	0.84	0.81
34.6 (optimal)	0.74	0.92	0.80
35.0	0.72	0.92	0.79
36.0	0.61	1.00	0.74
38.0	0.42	1.00	0.61
40.0	0.21	1.00	0.47

When age was added as a covariate, the predictive effect of CSA remained robust (OR 1.74 per degree, 95% CI 1.41-2.15, p < 0.001). Age showed only a marginal effect (OR 1.04 per year, 95% CI 1.00-1.08, p = 0.055). Model discrimination improved only minimally (AUC 0.885 vs. 0.881 without age), indicating that CSA alone accounted for most of the predictive information in distinguishing between normal and pathological shoulders.

**Figure 6:**
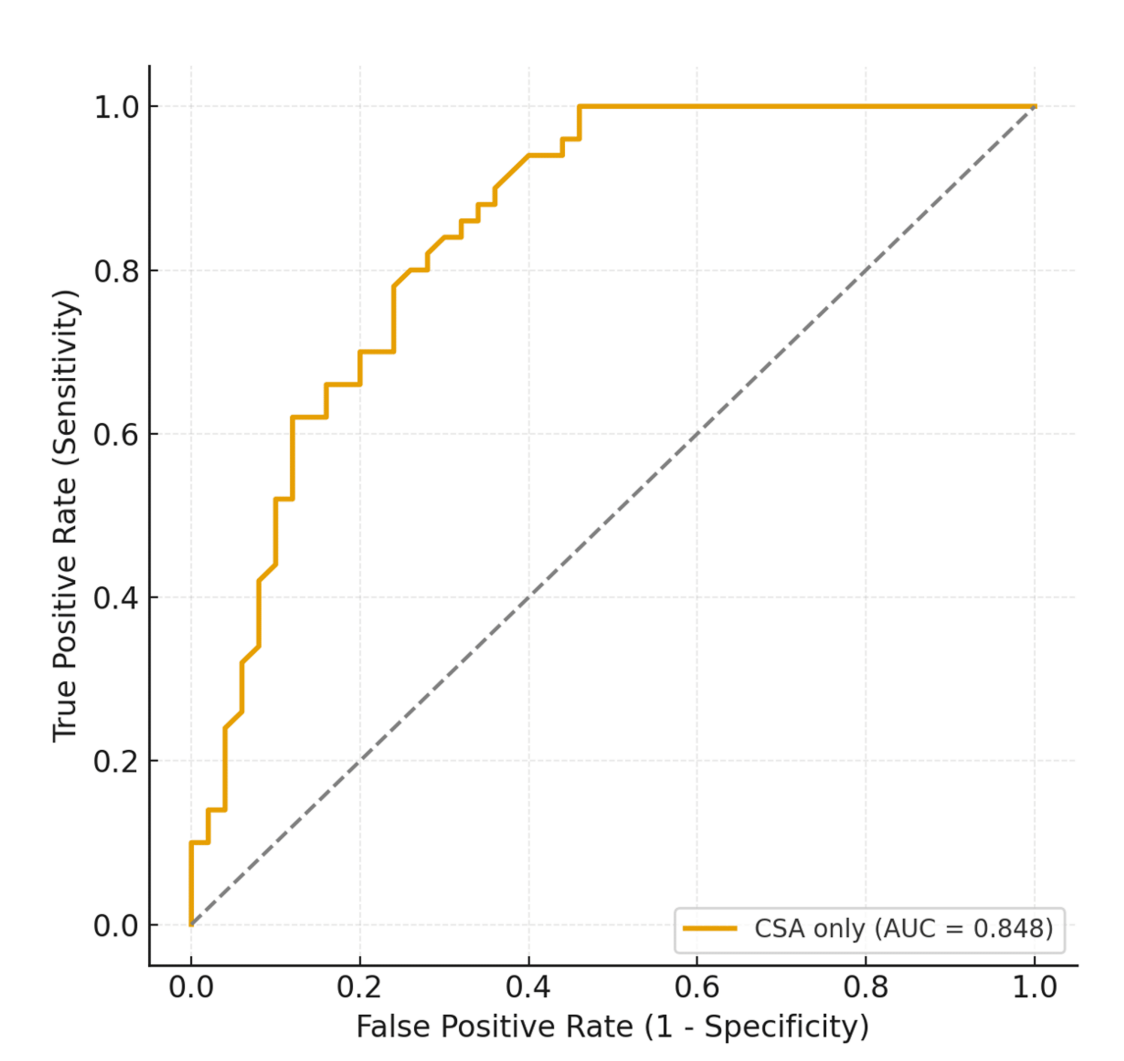
ROC curve for CSA predicting RCT among pathological shoulders. ROC: receiver operating characteristic; CSA: critical shoulder angle; RCT: rotator cuff tear; AUC: area under the curve.

Within the pathology cohort, CSA alone was a strong predictor of cuff tear. Logistic regression demonstrated that each additional degree of CSA increased the odds of cuff tear by 80% (OR 1.80, 95% CI 1.31-2.47, p < 0.001). Model discrimination was excellent (AUC 0.91; McFadden’s pseudo-R² 0.32). ROC analysis identified an optimal cutoff corresponding to a CSA of 36.5°, which achieved a sensitivity of 82% and a specificity of 86% (Figure 6). By contrast, applying CSA as a simple raw threshold at the same value resulted in slightly lower performance (sensitivity of 84% and specificity of 70%) (Table 5), underscoring the greater precision of the regression-based probability model compared with a crude angle split. Lower thresholds around 34°-35° provided very high sensitivity, supporting their utility in ruling out cuff tears.

**Table 5:** CSA cutoffs for RCT prediction - within pathology. CSA: critical shoulder angle; RCT: rotator cuff tear.

CSA cutoff (°)	Sensitivity	Specificity	Accuracy
34.0	1.00	0.40	0.70
35.0	0.98	0.54	0.76
36.0	0.88	0.66	0.77
36.5	0.84	0.70	0.77
36.9 (optimal)	0.78	0.76	0.77
38.0	0.66	0.82	0.74
40.0	0.34	0.92	0.63

When the GT morphology grade was added, predictive performance improved markedly, with excellent discrimination (AUC 0.92). CSA remained significant (OR 1.80 per degree, 95% CI 1.31-2.47, p < 0.001), while GT = 2 compared with GT = 1 increased the odds of cuff tear more than 10-fold (OR 10.4, 95% CI 2.6-41.4, p = 0.001). Applying stratified CSA thresholds by GT yielded optimal classification: CSA ≥ 40.6° for GT = 1 and CSA ≥ 34.7° for GT = 2, resulting in a sensitivity of 80%, specificity of 92%, and accuracy of 86%. A simplified clinical rule - ‘predict cuff tear if GT = 2 or CSA ≥ 38°’’ - provided higher sensitivity (88%) but with reduced specificity (74%).

**Figure 7:**
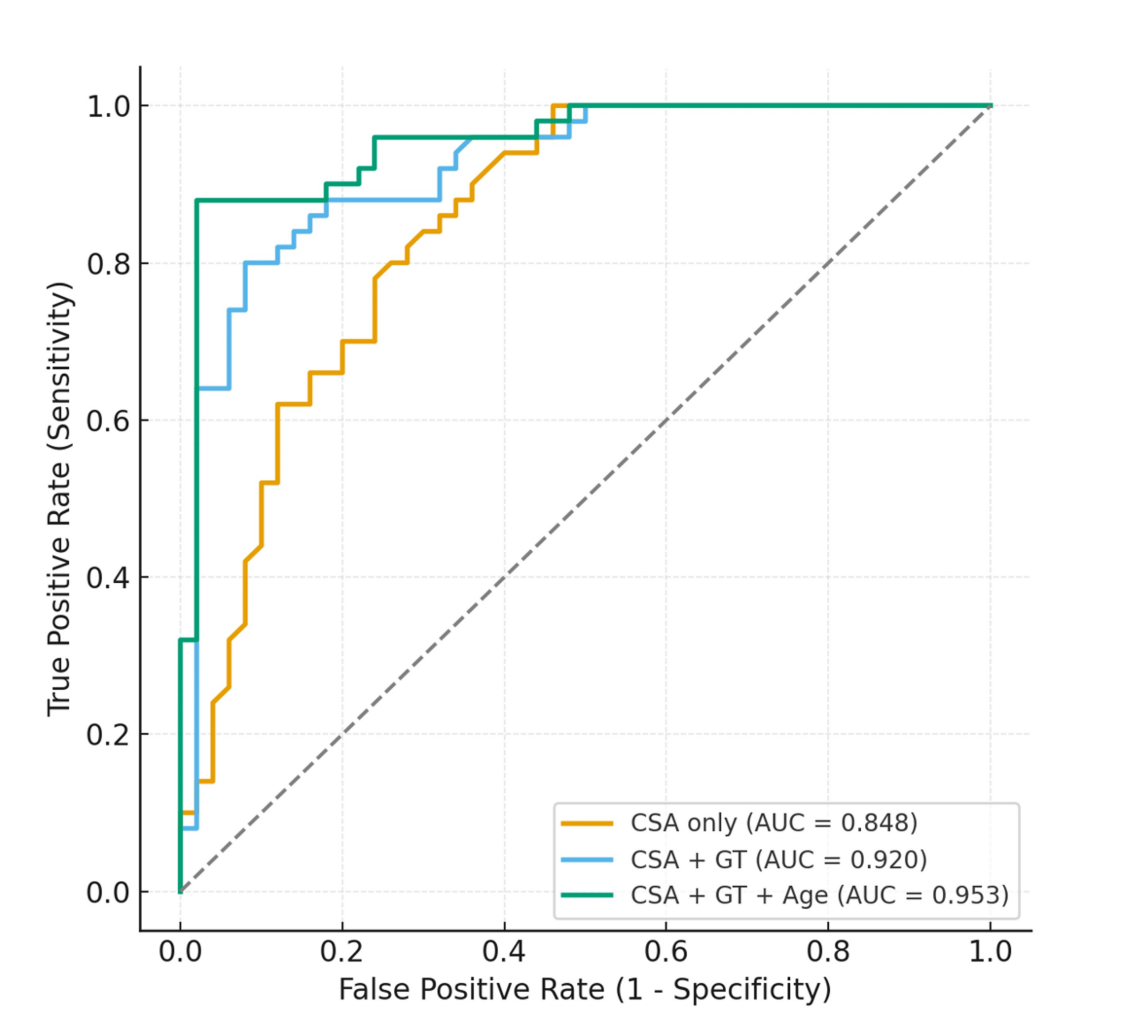
Receiver operating characteristic (ROC) curves for logistic regression models predicting cuff repair among pathological shoulders. The CSA-only model demonstrated good discrimination (AUC 0.85). Adding GT substantially improved performance (AUC 0.92), while the whole model, including CSA, GT, and age, provided the highest discrimination (AUC 0.95). CSA: critical shoulder angle; AUC: area under the curve; GT: greater tuberosity.

The inclusion of age further enhanced the model's explanatory power. Older age was independently associated with cuff tear (OR 1.19 per year, 95% CI 1.07-1.32, p = 0.002), while CSA and GT morphology remained significant predictors. Model discrimination remained excellent (AUC 0.95). Age- and GT-adjusted CSA thresholds provided the most accurate classification: at age 40 years, cuff tear was predicted if CSA ≥ 44.9° (GT = 1) or ≥41.0° (GT = 2); at age 50 years, thresholds were CSA ≥ 42.0° (GT = 1) or ≥38.0° (GT = 2); and at age 60 years, CSA ≥ 39.1° (GT = 1) or ≥35.1° (GT = 2). Applying these age-banded thresholds yielded a sensitivity of 80%, a specificity of 96%, and an overall accuracy of 88% (Table 6 and Figure 7).

**Table 6:** CSA cutoffs for RCT prediction for different models. CSA: critical shoulder angle; AUC: area under the curve; GT: greater tuberosity; RCT: rotator cuff tear.

Model	Predictors	AUC	Cutoff	Sensitivity	Specificity	Accuracy
CSA only	CSA	0.848	CSA ≥ 36.9° (~37°)	0.78	0.76	0.77
CSA + GT	CSA + GT	0.920	GT = 1 → CSA ≥ 40.6°; GT = 2 → CSA ≥ 34.7°	0.80	0.92	0.86
CSA + GT + age	CSA + GT + age	0.953	Age- and GT-adjusted CSA thresholds	0.80	0.96	0.88

**Figure 8:**
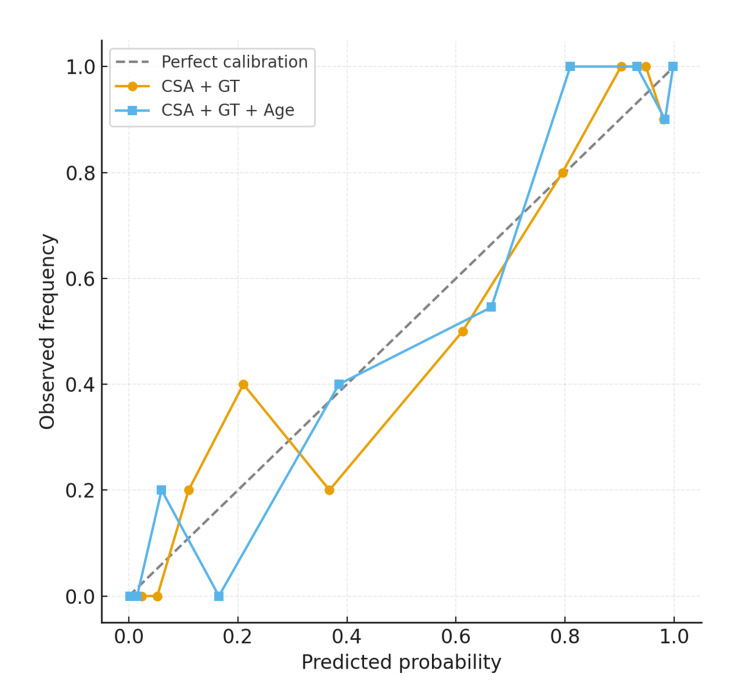
Calibration plots for logistic regression models predicting RCT within the pathology cohort. The CSA + GT model (orange circles) demonstrated good calibration with mild overprediction at higher risk levels. The full model, including CSA, GT, and age (blue squares), showed improved calibration, closely following the line of perfect prediction (dashed line). RCT: rotator cuff tear; CSA: critical shoulder angle; GT: greater tuberosity.

Calibration plots were used to assess the agreement between predicted probabilities and observed outcomes in the SAI vs. RCT models (Figure 8). The CSA + GT model demonstrated good overall calibration but showed a slight tendency to overestimate the probability of RCT at higher risk levels. On the other hand, the CSA + GT + age model showed a closer alignment with the line of perfect calibration, indicating a more reliable prediction of absolute risk across the full probability range.

## Discussion

This study found that CSA, GT morphology, and age were associated with RCT. When combined, these parameters improved predictive accuracy compared to CSA alone. We found normal shoulders to have an average CSA of 32.31° (SD ± 1.46), 34.82° (SD ± 3.15) associated with SAI, and 39.29° (SD ± 3.35) with RCTs. On average, 68% of RCTs were associated with GT irregularities or a bony chip (grade 2). A CSA cutoff of 34.6° differentiates normal from pathological shoulders (sensitivity 74%, specificity 92%, and accuracy 80%). CSA of 36.9° predicts RCTs with 77% accuracy, and each additional degree increases the probability by 80% (OR 1.80, 95% CI 1.31-2.47, p < 0.001). When GT morphology was added to CSA, performance improved markedly (AUC 0.92), while GT = 2 compared with GT = 1 increased the odds of cuff tear more than 10-fold (OR 10.4, 95% CI 2.6-41.4, p = 0.001). Adding the patient's age to the previous parameters further improved the discriminatory capabilities (AUC 0.95). Calibration plots demonstrated that a combination of CSA, GT morphology, and patient age can enhance absolute risk prediction for RCTs. This suggests that our combined model not only discriminates well but also provides reliable absolute risk estimates, an essential step toward clinical application.

Our findings align with Moor et al., who reported mean CSA values of ~38° in RCTs and confirmed CSA as the strongest scapular predictor of RCTs [1,2]. Studies showed that healthy populations have a CSA of ~33° [8]. Heuberer et al. demonstrated that a combination of CSA and age can predict multiple shoulder pathologies [9]. Our studies expanded on this by adding age. The biomechanical plausibility of our findings is supported by Gerber et al., who demonstrated that larger CSA values induce supraspinatus overload, and by Viehöfer et al., who showed that a larger CSA requires greater cuff activity to preserve stability [4,5]. Previous studies using CSA alone reported AUCs in the range of 0.70-0.85 for RCT prediction [10,11], whereas our combined model achieved an AUC of 0.95, demonstrating substantially higher discriminatory power.

Recent studies continue to demonstrate a clinically significant association between scapular morphology and cuff pathology. A 2024 systematic review and meta-analysis confirmed a significant correlation between larger CSA and the presence of RCTs across heterogeneous cohorts, reinforcing CSA as an extrinsic risk factor when measurement is standardised [12]. Beyond diagnosis, CSA also appears to be prognostic: a 2023 meta-analysis found that higher CSA values were associated with increased re-tear rates after arthroscopic repair, even though functional scores were not consistently worse, suggesting that morphology influences the risk of structural healing [13]. Methodological studies further support the reliability of CSA as a clinic-friendly metric, with excellent intra- and interobserver agreement reported using routine radiographs [14]. Collectively, these data corroborate our finding that CSA carries a substantial predictive signal that can be leveraged alongside GT changes and age.

In contrast, several reviews highlight that CSA alone is an imperfect discriminator, as results vary with radiographic technique, population, and alternative acromial indices [15,16]. Contemporary evidence around acromioplasty also remains mixed: while some analyses report that reducing CSA via acromioplasty is possible and may benefit selected type III acromion patients, others show no consistent improvement in long-term patient-reported outcomes, underscoring that morphology modification does not uniformly translate to clinical gain [17,18]. In this context, adjunct markers on plain films assume importance. Classic and more recent radiographic studies demonstrate that changes in the GT, such as sclerosis and marginal irregularity, are associated with cuff tears, showing sound sensitivity and specificity, although not perfect [19-21].

Our data align with this trajectory: integrating GT grade with CSA (and age) significantly improves discrimination and calibration compared to CSA alone, addressing a key limitation identified by prior CSA-centric literature. Despite extensive research on CSA, evidence is lacking regarding the integration of GT morphology [10,11]. GT irregularity may represent chronic tendon overload and structural adaptation, making it a biologically plausible marker of these conditions. This adds a layer of predictive accuracy beyond CSA alone. Our study adds GT morphology and age to CSA to strengthen predictive robustness.

To our knowledge, this is the first study to systematically evaluate CSA, GT morphology, and age in combination. We employed logistic regression and ROC analysis to further enhance clinical interpretability. Our study also employed practical clinical parameters, namely, plain radiographs and age, which further assist clinicians in expediting the diagnosis and planning patient management.

Our study has several limitations. First, this was a retrospective single-centre analysis. Generalisability requires multicentre multisurgeon analyses. Second, GT morphology can be somewhat subjective and unstandardised. Third, our study did not stratify risk by tear size, chronicity, or symptoms in relation to RCTs, which are areas that future research can focus on. Fourth, confirmation of findings included MRI/arthroscopy, which could limit generalisability to primary care/undiagnosed populations. Finally, radiographic positioning remains a confounder, despite standardisation.

Our study demonstrates that a simple model (CSA + GT + age) can be utilised as a triage tool to determine which patients require further investigation with advanced imaging (i.e., MRI scan). Additionally, this could streamline diagnostic pathways in primary and secondary care. Lastly, our model has the potential for use in risk stratification of asymptomatic patients with high-risk morphology.

Future research could focus on multicentre prospective validation of our findings. Incorporating automation/artificial intelligence (AI)-based CSA and GT measurements could save time and allow for a larger number of cohorts, in addition to ruling out inappropriate radiographs, thereby aiding in exclusion. Furthermore, focus could be diverted to investigating whether morphology-based risk stratification could guide prevention or early interventions.

## Conclusions

This study demonstrates that a simple, radiograph-based model incorporating CSA and GT morphology, along with patient age, can provide clinicians with meaningful assistance in identifying shoulder pathology at the first point of contact. This approach may streamline diagnostic pathways, reduce reliance on advanced imaging, and support earlier treatment decisions.

A CSA cutoff of 34.6° differentiated normal from pathological shoulders, while 36.9° distinguished SAI from RCTs. Incorporating GT morphology strengthened diagnostic confidence - patients with GT irregularity or a bony chip and CSA ≥ 34.7° had a 10-fold higher risk of RCT (AUC 0.92). Adding age further improved predictive accuracy (AUC 0.95; accuracy 88%), with lower CSA thresholds indicating higher RCT likelihood in older individuals (e.g., at 50 years, CSA ≥ 38.0°; at 60 years, CSA ≥ 35.1°). These findings support the integration of simple radiographic and demographic markers into early clinical assessment. Further multicentre validation is warranted to confirm generalisability across populations and imaging settings.
